# Characterization of Flagellar Propulsion of Soft Microrobotic Sperm in a Viscous Heterogeneous Medium

**DOI:** 10.3389/frobt.2019.00065

**Published:** 2019-07-31

**Authors:** Islam S. M. Khalil, Anke Klingner, Youssef Hamed, Veronika Magdanz, Mohamed Toubar, Sarthak Misra

**Affiliations:** ^1^Department of Biomechanical Engineering, University of Twente, Enschede, Netherlands; ^2^Department of Physics, The German University in Cairo, New Cairo, Egypt; ^3^Applied Zoology, Dresden University of Technology, Dresden, Germany; ^4^Department of Biomedical Engineering, University Medical Center Groningen, University of Groningen, Groningen, Netherlands

**Keywords:** flagellar propulsion, heterogeneous, magnetic, modeling, resistive-force theory, robotic sperm, Stokeslets

## Abstract

Several microorganisms swim by a beating flagellum more rapidly in solutions with gel-like structure than they do in low-viscosity mediums. In this work, we aim to model and investigate this behavior in low Reynolds numbers viscous heterogeneous medium using soft microrobotic sperm samples. The microrobots are actuated using external magnetic fields and the influence of immersed obstacles on the flagellar propulsion is investigated. We use the resistive-force theory to predict the deformation of the beating flagellum, and the method of regularized Stokeslets for computing Stokes flows around the microrobot and the immersed obstacles. Our analysis and experiments show that obstacles in the medium improves the propulsion even when the Sperm number is not optimal (*S*_*p*_ ≠ 2.1). Experimental results also show propulsion enhancement for concentration range of 0−5% at relatively low actuation frequencies owing to the pressure gradient created by obstacles in close proximity to the beating flagellum. At relatively high actuation frequency, speed reduction is observed with the concentration of the obstacles.

## 1. Introduction

Efficient propulsion on the microscale is one of the main targets of micro- and nanorobotics research. Various propulsion mechanisms have been demonstrated in the last decades (Nelson et al., 2010), including chemical propulsion, magnetic propulsion or biological propulsion by microorganisms (Behkam and Sitti, 2007) or sperm cells (Magdanz et al., 2013; Guix et al., 2014). Magnetic propulsion can be implemented with cork-screw-like motion of rigid helical swimmers (Ghosh and Fischer, 2009) or by magnetic actuation of soft, flexible rods (Dreyfus et al., 2005; Khalil et al., 2014; Williams et al., 2014). The magnetic actuation of flexible rods resembles the motion of spermatozoa, which move as pushers by bending waves traveling along their flagellum. Spermatozoa are biological microswimmers that have evolved to swim efficiently through complex environments of the reproductive tract (Gaffney et al., 2011). On their way to the fertilization site, mammalian sperm cells migrate through fluids with a wide range of viscosities, pH, and complex compositions of macromolecules and cells. Increased viscosity imposes an increased resistance to progression on the microswimmer and requires an increased energy output (Kirkman-Brown and Smith, 2011). Spermatozoa also undergo different beat patterns and transitions between planar and helical flagellar propulsion to maintain relatively high speed regardless to the rheological and physical properties of the background fluid (Kantsler et al., 2014; Li and Ardekani, 2015; Khalil et al., 2018b). Motile bacteria have shown increased swimming velocity in increased viscosity due to the interaction with the fibrous network. This network allows the microorganisms to push themselves off the surrounding obstacles, increasing the pitch of the helical motion (Berg and Turner, 1979; Leshansky, 2009; Ullrich et al., 2016). It is also known that geometrical swimming through an array of obstacles can lead to an increased swimming speed in colloidal suspensions (Munch et al., 2016). Nelson and Peyer have also characterized artificial bacterial flagella (ABFs) in water and solutions of methyl cellulose at different concentrations (Nelson and Peyer, 2014). They have demonstrated that ABFs show similar behavior to microgroganisms as the effective pitch increases with the concentration of the solution.

**Figure 1:**
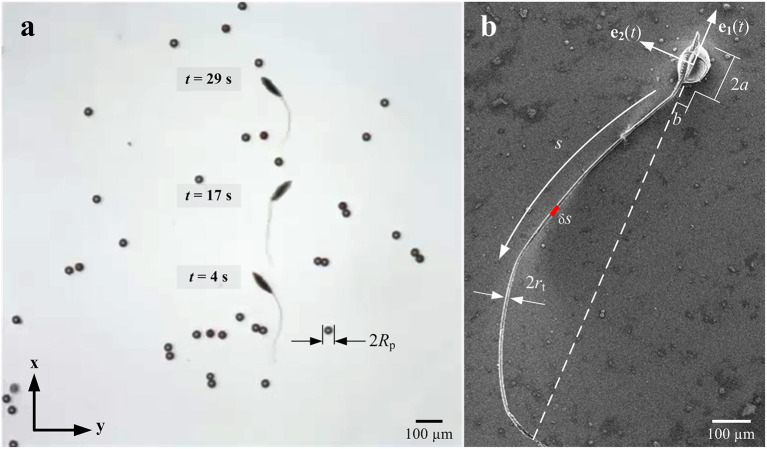
A soft microrobotic sperm swims in a viscous heterogeneous medium under the influence of a periodic magnetic field. **(a)** The medium (glycerin with viscosity of 0.95 Pa.s) contains particles with average diameter of 30 μm and concentration of 3.2%. **(b)** Scanning electron microscopy image of a soft microrobotic sperm shows its magnetic head and ultra-thin flexible tail. **e**_1_(*t*) and **e**_2_(*t*) are orthonormal vectors of the material frame of the microrobotic sperm. The magnetic dipole moment of the head is oriented along **e**_1_(*t*) and enables directional control under the influence of external magnetic fields.

The ability to actuate artificial microswimmers in complex environments is likely to be an important advancement toward their translation into *in vivo* applications. Therefore, the propulsion of artificial microswimmers have to be tested in *in vivo* scenarios, such as complex and crowded environments, and along and against the flowing streams of the medium (Khalil et al., 2016a). Also other body fluids, such as blood, can be conceived as complex, colloidal suspensions of high viscosity due to the relatively high amount of cells in the fluid. Ullrich et al. have investigated the helical propulsion of rigid helical microrobots in fibrous environments with various collagen fiber concentration, and observed propulsion enhancement due to movements similar to corkscrew motion without slippage (Ullrich et al., 2016). The locomotion of rigid helical robots has been also experimentally studied in tissue via the action of a uniform and non-uniform rotating magnetic fields (Mahoney et al., 2012; Nelson et al., 2013). However, locomotion of externally actuated soft microrobots has not been investigated in a complex and crowded mediums similar to environments encountered *in vivo*. Espinosa-Garcia et al. have investigated experimentally the impact of the fluid elasticity on the flagellar propulsion of flexible swimmers (Espinosa-Garcia et al., 2013). They have shown that the propulsion is systematically enhanced by the elasticity of the fluid. In this work, we mimic such complex environment by immersing soft microrobotic sperm samples, fabricated by electrospinning as described in Khalil et al. (2016b), in a highly viscous solution (glycerin) in the presence of spherical particles, as shown in Figure 1a. We investigate the influence of the concentration of these particles in the colloidal suspension on the swimming speed theoretically and experimentally.

## 2. Modeling of Soft Microrobotic Sperm in a Viscous Heterogeneous Medium

A soft microrobotic sperm swims by a beating flagellum in low Reynolds numbers. The flagellar wave propagation along its tail is achieved by exerting a periodic magnetic torque on the magnetic head of the microrobotic sperm. In the case of a medium with immersed particles, the elastic tail of the microrobotic sperm interacts with the surrounding fluid and the immersed particles. The resistive-force theory (RFT) is implemented to predict the influence of the particles on the deformation of the elastic tail and the flagellar propulsion.

### 2.1. Flagellar Propulsion Using the Resistive-Force Theory

**Figure 2:**
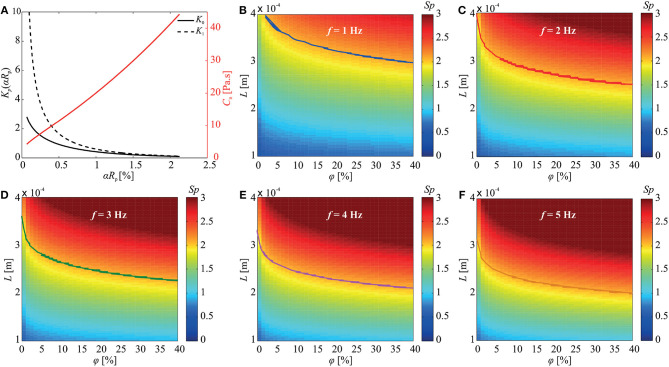
The concentration (φ) and size (*R*_p_) of the immersed obstacle influence the normal drag coefficient (*c*_n_) and the Sperm number (*Sp*) of the soft microrobotic sperm. **(A)** The normal drag coefficient is calculated using the modified Bessel functions *K*_0_(α*R*_p_) and *K*_1_(α*R*_p_) based on (2). **(B–F)** The influence of the concentration of the immersed particles and the length (*L*) of the tail on the Sperm number is calculated for 1 ≤ *f* ≤ 5 Hz. The curves represent the combinations of φ and *L* that achieve *Sp* = 2.1. Parameters: 2*a* = 98 μm, 2*b* = 31 μm, 2*r*_t_ = 8 μm, *L* = 284 μm, *E* = 0.58 GPa, and μ = 0.95 Pa.s.

The soft microrobotic sperm consists of a prolate spheroidal head of length 2*a* and radius *b* (Figure 1b). The head is rigidly attached to an ultra-thin flexible tail of bending stiffness κ, length *L*, and diameter 2*r*_t_. The microrobots are allowed to swim in a medium with viscosity μ, characterized by low Reynolds numbers hydrodynamics (*Re* = ρ*v*_*x*_(*L* + 2*a*)/μ) on the order of O(10^−5^), where ρ is the density of the medium and *v*_*x*_ is the swimming speed. The medium contains randomly distributed and isotropic spherical particles with an average diameter 2*R*_p_ and concentration $\varphi =\pi R_{\text{p}}^{2}N/A$, where *N* is the number of particles within an area *A*. The particles are spherical and the drag force exerted (**F** = 6π*μR*_p_**u**) on a single particle times the number density ($n=\varphi /\frac{4}{3}\pi R_{\text{p}}^{3}$) of particles is equal to the mean resistance force per unit volume, *n***F** = μα^2^**u**, where **u** and α are the velocity field vector and a frequency parameter, respectively (Leshansky, 2009). The random spare array-based model of Leshansky also assumes a sufficiently large spacing between the obstacles to consider that the swimmer does not distort the matrix of particles. We analyze the motion in two-dimensional space on the medium-air interface to enable the microrobotic sperm and the particles to lie on the same plane. The samples are fabricated by electrospinning a solution of polystyrene in dimethylformamide and magnetic particles. During electrospinning, the magnetic particles are embedded into the polymer matrix of the spheroidal head and provide an average magnetic moment **M**. This magnetic moment enables the soft microrobotic sperm to align along external magnetic field lines. A homogenous magnetic field **B** with a sinusoidally varying orthogonal components at frequency ω induces a bending wave along the tail. The soft microrobotic sperm samples have a symmetric geometry with respect to their propulsion axis (Figure 1). Therefore, the component of the surface tension force along the lateral axis of the microrobot are equal and act along opposite directions. Therefore, the surface tension does not have major influence on the propulsion. The deformation of the tail is governed by

(1) $$\begin{array}{r} \kappa \frac{\partial^{4}y}{\partial x^{4}}\left(x,t\right)+c_{\text{n}}\left(\alpha \right)\frac{\partial y}{\partial t}\left(x,t\right)=0, \end{array}$$

where *y*(*x, t*) describes the deformation of the flexible tail, relative to a fixed frame of reference (**e**_1_(*t*), **e**_2_(*t*)), where **e**_1_(*t*) and **e**_2_(*t*) are orthonormal vectors such that **e**_1_(*t*) is oriented along the long axis of the head. Further, *c*_n_ is the following normal drag coefficient (Leshansky, 2009):

(2) $$\begin{array}{r} c_{\text{n}}\left(\alpha \right)=4\pi \mu \left(\frac{1}{4}{\left(\alpha R_{\text{p}}\right)}^{2}+\alpha R_{\text{p}}\frac{K_{1}\left(\alpha R_{\text{p}}\right)}{K_{0}\left(\alpha R_{\text{p}}\right)}\right), \end{array}$$

where *K*_*p*_(α*R*_p_) is the modified Bessel function of degree *p* (for *p* = 0, 1) and $\alpha^{2}=\frac{9\varphi }{2R_{\text{p}}^{2}}$. Equation (2) indicates that the normal drag coefficient is influenced by the size and concentration of the immersed particles (Figure 2A), and thus the deformation of the elastic tail is also affected. In addition, the Sperm number is also affected by the concentration of the particles in the medium, and as a consequence, the propulsive force is influenced by the size and concentration of the immersed particles. This relation can be shown by the Sperm number of the soft microrobotic sperm given by

(3) $$\begin{array}{r} Sp=L{\left(\frac{8\omega c_{\text{n}}}{r_{\text{t}}^{4}E}\right)}^{1/4}, \end{array}$$

where *E* is the Young's modulus of the microrobot. The Sperm number provides a measure of the propulsive force for planar flagellar propulsion. An optimal propulsive force is generated at *Sp* ≈ 2.1 (Yu et al., 2006). Figures 2B–F show the Sperm number vs. the concentration of the particles and length of the elastic tail for actuation frequencies of 1–5 Hz. This simulation indicates combinations of φ and *L* (shown by the solid curves) that achieves *Sp* ≈ 2.1 to enhance the propulsion.

The tangential drag coefficient (*c*_t_) is also affected by the concentration and size of the immersed particles and is calculated using

(4) $$\begin{array}{r} c_{\text{t}}\left(\alpha \right)=4\pi \mu \left(\frac{1}{2}\alpha R_{\text{p}}\frac{K_{1}\left(\alpha R_{\text{p}}\right)}{K_{0}\left(\alpha R_{\text{p}}\right)}\right). \end{array}$$

The normal and tangential drag coefficients provide an anisotropic operator that relates the hydrodynamic drag force exerted on a segment (δ*s*) to its velocity. Therefore, we use the drag coefficients (2) and (4) to calculate the exerted force δ*F*_f_ on δ*s* as follows (Gray and Hancock, 1955):

(5) $$\begin{array}{r} \delta F_{\text{f}}=\frac{\left(c_{\text{n}}-c_{\text{t}})v_{y}\frac{\text{d}y}{\text{d}x}-v_{x}(c_{\text{t}}+c_{\text{n}}{\left(\frac{\text{d}y}{\text{d}x}\right)}^{2}\right)}{1+{\left(\frac{\text{d}y}{\text{d}x}\right)}^{2}}\delta s, \end{array}$$

where *v*_*y*_ is the transverse velocity component of the segment δ*s* (Figure 1b), respectively. Further, $\frac{\text{d}y}{\text{d}x}$ is the orientation of the segment with respect to the propulsion axis. The total thrust force exerted by the flexible tail must balance the drag of the head using

(6) $$\begin{array}{r} \int_{0}^{L}\frac{\left(c_{\text{n}}-c_{\text{t}}\right)v_{y}\frac{\text{d}y}{\text{d}x}-v_{x}\left(c_{\text{t}}+c_{\text{n}}{\left(\frac{\text{d}y}{\text{d}x}\right)}^{2}\right)}{1+{\left(\frac{\text{d}y}{\text{d}x}\right)}^{2}}\text{d}x-6\pi \mu {\left(ab^{2}\right)}^{1/3}v_{x}=0. \end{array}$$

In contrast to sperm cells and flagellated microorganisms, our soft microrobotic sperm depends on an external magnetic field with a sinusoidally varying orthogonal component to achieve flagellar propulsion. This magnetic field exerts a magnetic torque (**M** × **B**) on its magnetic head. Therefore, the boundary conditions at the proximal end of the flexible tail are *y*(0, *t*) = *b* sin α sin(ω*t*) and ∂*y*(0, *t*)/∂*x* = tan α sin(ω*t*), where tan α = d*y*/d*x* is the orientation of δ*s* with respect to **e**_1_(*t*). The distal end is not subject to magnetic force and torque. Therefore, the boundary conditions at the distal end of the flexible tail are ∂^2^*y*(*L, t*)/∂*x*^2^ = 0 and ∂^3^*y*(*L, t*)/∂*x*^3^ = 0. The magnetic field **B** is generated using an array of four orthogonal electromagnetic coils to control the direction of the microrobotic sperm in two-dimensional space and achieve flagellar propulsion. This propulsion results in a local flow-field in the background fluid.

### 2.2. Stokeslets Flow-Fields

The fluid surrounding the soft microrobotic sperm and the immersed particles are influenced by the beating tail. The governing fluid mechanics for a soft microrobotic sperm in low Reynolds numbers are given by the following Stokes equation:

(7) $$\begin{array}{rcl} \mu \nabla^{2}\text{u}+\text{f}-\nabla p & = & 0, \end{array}$$

(8) $$\begin{array}{rcl} \nabla \cdot \text{u} & = & 0, \end{array}$$

where **u** is the velocity field vector, **f** and *p* are the body force of the soft microrobotic sperm on the fluid and the scalar pressure field, respectively. We assign *N* Stokeslets boundary points on the surface of the microrobotic sperm and the surface of the immersed particles in the medium. These particles are randomly arranged within the vicinity of the microrobotic sperm and have zero initial velocity. The pressure caused by a force **f**_*k*_ at a point **x**_*k*_ along the flexible tail is approximated by (Cortez, 2001; Khalil et al., 2019)

(9) $$\begin{array}{r} p\left(\text{x}\right)=\overset{N}{\underset{k=1}{\sum }}\frac{1}{2\pi }\left[{\text{f}}_{k}\cdot \left(\text{x}-{\text{x}}_{k}\right)\right]\left(\frac{r_{k}^{2}+2\epsilon^{2}+\epsilon \sqrt{r_{k}^{2}+\epsilon^{2}}}{\left(\sqrt{r_{k}^{2}+\epsilon^{2}}+\epsilon \right){\left(r_{k}^{2}+\epsilon^{2}\right)}^{3/2}}\right), \end{array}$$

where *r*_*k*_ = |**x** − **x**_*k*_| and ϵ is a parameter that describes the sharpness of a delta-function. This delta function approximates the forces exerted by the beating tail on the fluid. The velocity field, due to force **f**_*k*_ at points **x**_*k*_, is given by

(10) $$\begin{array}{r} \text{u}\left(\text{x}\right)=\frac{-{\text{f}}_{k}}{2\pi \mu }\left[\ln\left(\sqrt{r_{k}^{2}+\epsilon^{2}}+\epsilon \right)-\frac{\epsilon \left(\sqrt{r_{k}^{2}+\epsilon^{2}}+2\epsilon \right)}{\left(\sqrt{r_{k}^{2}+\epsilon^{2}}+\epsilon \right)\sqrt{r_{k}^{2}+\epsilon^{2}}}\right]\\ +\frac{1}{4\pi \mu }\left[{\text{f}}_{k}\cdot \left(\text{x}-{\text{x}}_{k}\right)\right]\left(\text{x}-{\text{x}}_{k}\right)\left[\frac{\sqrt{r_{k}^{2}+\epsilon^{2}}+2\epsilon }{\left(\sqrt{r_{k}^{2}+\epsilon^{2}}+\epsilon \right){\left(r_{k}^{2}+\epsilon^{2}\right)}^{3/2}}\right]. \end{array}$$

**Figure 3:**
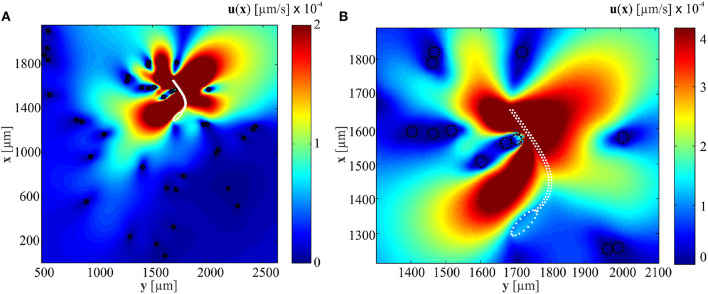
A soft microrobotic sperm achieves flagellar propulsion in a medium with immersed particles (black circles). **(A)** The microrobotic sperm swims at an average speed of 104 μm/s at frequency of 1 Hz. The Stokeslets flow-fields are calculated using (2.2) and the white dashed line represents the boundaries of the soft microrobotic sperm. **(B)** The velocity of the fluid past each obstacle (in the direction opposite to the flagellum) decreases by ~3 orders of magnitude. The flagellum pushes the soft microrobot off the surrounding particles.

Equation (2.2) can be used to calculate the velocity field given the force exerted by the flexible tail on the surrounding fluid. It can also be used to calculate the necessary forces **f**_*k*_ at Stokeslets points to initiate the given velocities **u**_*k*_ at positions **x**_*k*_. Figure 3A shows the influence of immersed particles on the flow-field created by a soft microrobotic sperm at particle concentration of 3.2% and actuation frequency of 1 Hz. The positions of the particles and the soft microrobotic sperm are determined experimentally (Figure 1a). Elastic theory based on (1) is used to determine the tail deformation and velocities *v*_*y*_ = d*y*/d*t* of the Stokelet points on the tail. Additional translation speed is chosen as 110 μm/s based on the experiments. The distance between Stokeslet points is d*s* = 2*r*_t_. The sharpness of the delta function is ϵ = 0.25d*s*. The flow-field (close to the beating flagellum, as shown in Figure 3B) indicates that the velocity of the medium past each obstacle is decreased by 3 orders of magnitude. Therefore, the presence of these particles hinders the flow of the medium by the beating flagellum. As a consequence, a local pressure gradient is created within the vicinity of the flagellum and the propulsion is influenced based on (7)–(9). Figure 3 also shows that the velocity field created by the beating flagellum is relatively greater than that of the wiggling head. Therefore, the pressure gradient in close proximity to the distal tip of the flagellum is higher than the pressure close to the head, and results in propulsion enhancement. In the case of relatively higher actuation frequencies, the amplitude of the beating flagellum decreases with the increasing frequency. The dependence of the amplitude on the actuation frequency indicates that the propulsion enhancement is likely to be achieved at relatively low beating frequencies.

### 2.3. Numerical Scheme of the Hydrodynamic Model

The deformation of the tail and the drag forces are determined using finite-difference discretization of (1). The tail of the soft microrobotic sperm is discretized into *N* = 100, equally spaced mesh nodes. The partial differential Equation (1) is solved numerically. The time-dependent trajectory of the soft microrobotic sperm is calculated by forward Euler integration over consecutive time-steps of Δ*t* = 1 × 10^−3^ s. The tail deformation (*y*(*x, t*)) is represented as

(11) $$\begin{array}{r} y\left(x,t\right)=\left[\begin{array}{cccc} y_{1} & y_{2} & \ldots & y_{N} \end{array}\right]\;\text{and }y\left(x,t-\Delta t\right)=\left[\begin{array}{cccc} {\tilde{y}}_{1} & {\tilde{y}}_{2} & \ldots & {\tilde{y}}_{N} \end{array}\right]. \end{array}$$

The boundary conditions provide *y*_1_ = *b* sin α sin(ω*t*) and *y*_2_ = *y*_1_ + Δ*x* tan α sin(ω*t*). In addition, ${\tilde{y}}_{2}=\tan\alpha \sin\left(\omega t\right)$, and *y*_2_ = *y*_1_ + tan α sin (ω(*t* + Δ*t*)]). The *i*th first- and second-order derivatives of the deformation are approximated using

(12) $$\begin{array}{r} \frac{\partial y}{\partial x}{|}_{i+1}=\frac{y_{i+1}-y_{i}}{\Delta x}\;\text{and }\frac{\partial^{2}y}{\partial x^{2}}{|}_{i}=\frac{y_{i+1}-2y_{i}+y_{i-1}}{\Delta x^{2}}\;\\ \text{for }\left(i=3,\ldots ,N\right). \end{array}$$

Similarly, the *i*th third- and fourth-order derivatives are approximated by

(13) $$\begin{array}{r} \frac{\partial^{3}y}{\partial x^{3}}{|}_{i+1}=\frac{y_{i+2}-3y_{i+1}+3y_{i}-y_{i-1}}{\Delta x^{3}}\;\text{and }\frac{\partial^{4}y}{\partial x^{4}}{|}_{i}\\ =\frac{y_{i+2}-4y_{i+1}+6y_{i}-4y_{i-1}+y_{i-2}}{\Delta x^{4}}. \end{array}$$

Finally, Equations (12) and (13) are arranged in a system of *N* equations for *N* unknowns in the following form:

(14) $$\begin{array}{r} \left(\begin{array}{cccccccc} 1 & 0 & 0 & 0 & 0 & 0 & \ldots & 0\\ -1 & 1 & 0 & 0 & 0 & 0 & \ldots & 0\\ 1 & -4 & 6+\ell & -4 & 1 & 0 & \ldots & 0\\ 0 & 1 & -4 & 6+\ell & -4 & 1 & \ldots & 0\\ \vdots & \vdots & \ddots & \ddots & \ddots & \ddots & \ddots & \vdots \\ 0 & 0 & \vdots & 1 & -4 & 6+\ell & -4 & 1\\ 0 & 0 & \ldots & 0 & -1 & 3 & -3 & 1\\ 0 & 0 & \ldots & 0 & 0 & 1 & -2 & 1 \end{array}\right)\left(\begin{array}{c} y_{1}\\ y_{2}\\ y_{3}\\ y_{4}\\ \vdots \\ y_{N-2}\\ y_{N-1}\\ y_{N} \end{array}\right)=\left(\begin{array}{c} b\sin\alpha \sin\left(\omega t\right)\\ \Delta x\tan\alpha \sin\left(\omega t\right)\\ \ell {\tilde{y}}_{3}\\ \ell {\tilde{y}}_{4}\\ \vdots \\ -\ell {\tilde{y}}_{N-2}\\ 0\\ 0 \end{array}\right), \end{array}$$

where ℓ is given by

(15) $$\begin{array}{r} \ell =\frac{c_{\text{n}}\Delta x^{4}}{\kappa \Delta t}. \end{array}$$

**Figure 4:**
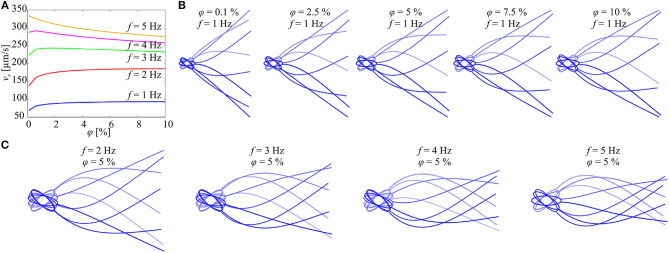
Forward speed (*v*_*x*_) of the soft microrobotic sperm is calculated vs. the concentration (φ) of the immersed particles for actuation frequency range of 1 ≤ *f* ≤ 5 Hz. Parameters: 2*a* = 98 μm, 2*b* = 31 μm, 2*r*_t_ = 8 μm, *L* = 284 μm, and μ = 0.95 Pa.s. **(A)** At relatively low actuation frequencies, the swimming speed increases with the concentration. For *f* ≥ 3 Hz, the speed decreases with the concentration. **(B)** Flagellar shapes are calculated for 0.1 ≤ φ ≤ 10% and *f* = 1 Hz. **(C)** Flagellar shapes are calculated for 2 ≤ *f* ≤ 5 Hz and φ = 5%.

The initial configuration of the elastic tail is set to a straight line along the propulsion axis **e**_1_(*t*). The tail deformation is calculated for several beat cycles, and the last five beat cycles are used in the calculation of the time-dependent forward speed (using 6) to mitigate the transient response. Figure 4 shows the forward speed and the flagellar shapes vs. the concentration of the particles and the actuation frequency. At *f* = 1 Hz and *f* = 2 Hz, the swimming speed of the microrobotic sperm is enhanced with the concentration. As the actuation frequency increases, we observe that the concentration of the particles has a negligible effect on the propulsion (Figure 4A) and for *f* ≥ 4 the speed decreases with the concentration. Figure 4B shows the flagellar shapes for 0.1 ≤ φ ≤ 10% and *f* = 1 Hz. The deformation of the beating tail at the distal end decreases with the actuation frequency, as shown in Figure 4C. Therefore, the flow-field and pressure gradient exerted on the particles decrease with the actuation frequency and do not result in propulsion enhancement.

## 3. Characterization of Flagellar Propulsion in a Viscous Heterogeneous Medium

Frequency response of soft microrobotic sperm samples is studied using an electromagnetic system under microscopic guidance, for various concentrations and random initial positions of the immersed particles.

### 3.1. System Description

The soft microrobotic sperm samples are prepared by electrospinning a solution of polystyrene (168 N, BASF AG) in dimethylformamide (DMF) and magnetic particles with average diameter of 30 μm. The polymer concentration is 25 wt % in DMF and the weight ratio of the iron to polystyrene is 1:2. The solution is injected using a syringe pump at flow rate of 20 μl/min under the influence of an applied electric potential with electric gradient of 100 kV/m. This electric potential is applied between the syringe and a collector and beaded-fibers are fabricated and cut to provide soft microrobotic sperm samples, as shown in Figure 1b. The average modulus of elasticity (*E*) of our samples is 0.58 ± 0.054 GPa, and is characterized by depth sensing indentation (Khalil et al., 2018a). The soft microrobotic samples are contained in a deep chamber with glycerin of viscosity 0.95 Pa.s. The chamber also contains particles (blue polystyrene particles, Micromod Partikeltechnologie GmbH, Rostock-Warnemuende, Germany) with average diameter of 30 μm. The soft microrobotic sperm samples are actuated using an orthogonal configuration of electromagnetic coils that surrounds the chamber. Each electromagnetic coil has an inner-, outer-diameter, and length of 20, 40, and 80 mm, respectively. The wire thickness is 0.7 mm and each coil has 3,200 turns, and the coil generates maximum magnetic field of 70 mT in the common center of the electromagnetic configuration. The swimming speed and the tail deformation of the soft microrobotic sperm samples are observed using a microscopic unit (MF Series 176 Measuring Microscopes, Mitutoyo, Kawasaki, Japan), and videos are acquired using a camera (avA1000-120kc, Basler Area Scan Camera, Basler AG, Ahrensburg, Germany) and a 10× Mitutoyo phase objective.

### 3.2. Frequency Response Characterization

**Figure 5:**
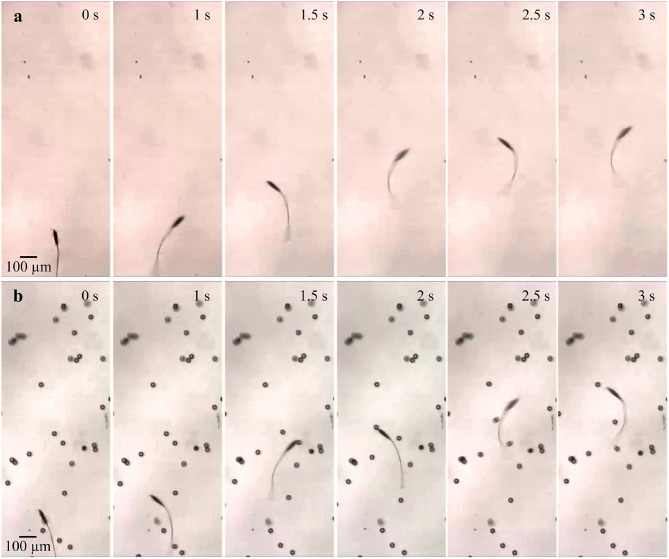
Image sequence of a soft microrobotic sperm demonstrating flagellar propulsion inside a viscous medium with and without spherical particles. The forward swimming speed (*v*_*x*_) is measured under the influence of actuation frequency of 1 Hz. 2*b* = 23.1 μm, 2*a* = 90.5 μm, and *L* = 260 μm. **(a)** At φ = 0%, *v*_*x*_ = 103 μm/s. **(b)** At φ = 3%, *v*_*x*_ = 115.8 μm/s.

**Figure 6:**
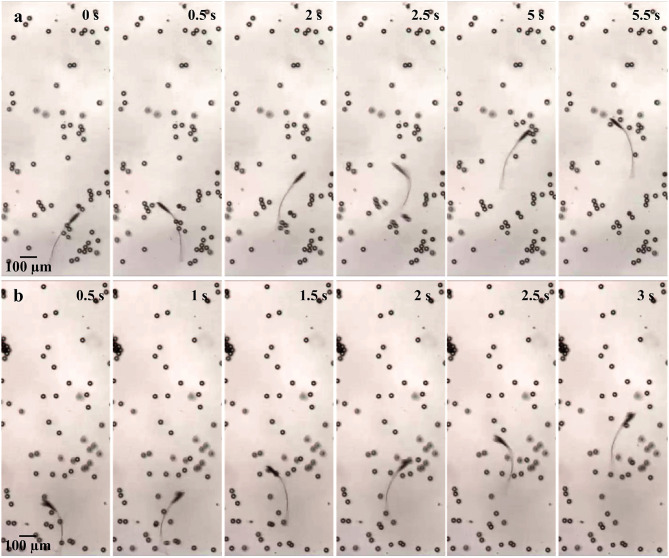
Image sequence of a soft microrobotic sperm demonstrating flagellar propulsion inside a viscous medium with spherical particles at actuation frequencies of 1 and 2 Hz. The forward swimming speed (*v*_*x*_) is measured at concentration (φ) of 7%. 2*b* = 23.1 μm, 2*a* = 90.5 μm, and *L* = 260 μm. **(a)** At *f* = 1 Hz, *v*_*x*_ = 124.1 μm/s. **(b)** At *f* = 2 Hz, *v*_*x*_ = 149.8 μm/s.

The frequency response of the soft microrobotic sperm samples is characterized in the absence and presence of immersed spherical particles with average diameter of 30 μm. The area concentration is varied between 0 and 10%. In each trial, the soft microrobotic sperm is allowed to achieve flagellar propulsion for the mentioned concentration range and under the influence of oscillating magnetic fields with frequency range between 1 and 5 Hz. The frequency response is limited to this range owing to the step-out frequency of the microrobotic sperm samples (above 5 Hz). The initial position of the immersed spherical particles is influenced after each trial due to the induced flow-field by the microrobotic sperm. Therefore, the average forward speed is measured vs. the average concentration of the immersed particles. In each experiment, the soft microrobotic sperm is allowed to swim in the absence of particles, as shown in Figure 5a. The same sample is also used to measure the swimming speed in the presence of particles. Figure 5b shows the response of the microrobotic sperm (2*b* = 23.1 μm, 2*a* = 90.5 μm, and *L* = 260 μm) under the influence of actuation frequency of 1 Hz in a medium with concentration of 3%. The average swimming speed is 103 and 115.8 μm/s for φ = 0% and φ = 3%, respectively. Figures 6a,b show the response of the same microrobotic sperm in a medium with concentration of 7% under the influence of actuation frequencies of 1 Hz and 2 Hz, respectively. At *f* = 1 Hz and *f* = 2 Hz the average swimming speed is measured as 124.1 and 149.8 μm/s, respectively.

**Figure 7:**
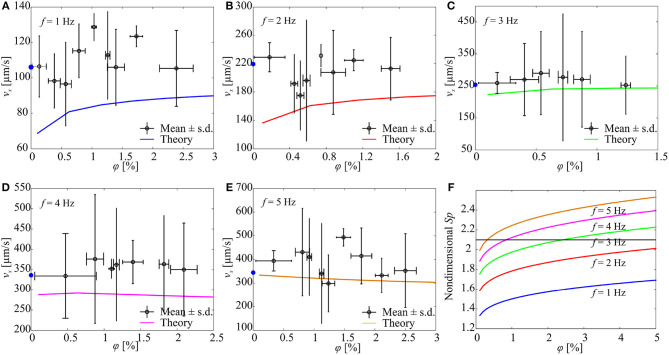
The forward speed (*v*_*x*_) of a soft microrobotic sperm sample is characterized vs. the concentration (φ) of spherical particles and actuation frequency (*f*) of the magnetic field. 2*a* = 98 μm, 2*b* = 31 μm, 2*r*_t_ = 8 μm, *L* = 284 μm. The blue circles indicate the average speed of the microrobot in the absence of particles. Each data point represents the average swimming speed of five trials at each concentration and actuation frequency. **(A)** *v*_*x*_ for *f* = 1 Hz. **(B)** *v*_*x*_ for *f* = 2 Hz. **(C)** *v*_*x*_ for *f* = 3 Hz. **(D)** *v*_*x*_ for *f* = 4 Hz. **(E)** *v*_*x*_ for *f* = 5 Hz. **(F)** The concentration of the particles influences the Sperm number and the propulsive force of the microrobot. *S*_*p*_ is calculated using (3).

**Figure 8:**
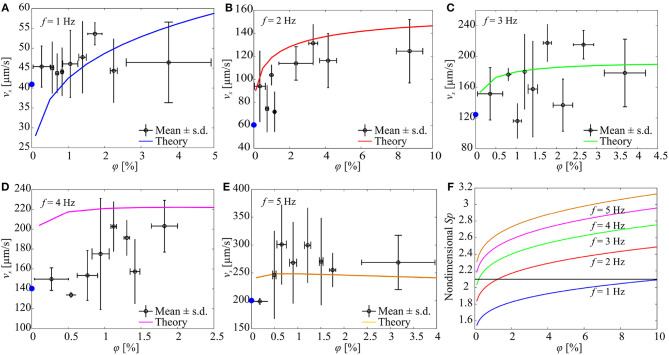
The forward speed (*v*_*x*_) of a soft microrobotic sperm sample is characterized vs. the concentration (φ) of spherical particles and actuation frequency (*f*) of the magnetic field. 2*a* = 125 μm, 2*b* = 44 μm, 2*r*_t_ = 8 μm, *L* = 346 μm. The blue circles indicate the average speed of the microrobot in the absence of particles. Each data point represents the average swimming speed of five trials at each concentration and actuation frequency. **(A)** *v*_*x*_ for *f* = 1 Hz. **(B)** *v*_*x*_ for *f* = 2 Hz. **(C)** *v*_*x*_ for *f* = 3 Hz. **(D)** *v*_*x*_ for *f* = 4 Hz. **(E)** *v*_*x*_ for *f* = 5 Hz. **(F)** The concentration of the particles influences the Sperm number and the propulsive force of the microrobot. *S*_*p*_ is calculated using (3).

Figure 7 shows the response of a microrobotic sperm with a major head diameter of 98 μm, minor head diameter of 31 μm, and tail length of 284 μm. At actuation frequency of 1 Hz, the average forward speed increases with the concentration of the immersed particles, as shown in Figure 7A. Each data point represents the average swimming speed of five trials at each concentration and actuation frequency. In each trial, the microrobot is allowed to swim within a range of 6–8 body lengths and the position of its head is tracked, and the speed is calculated by numerical differentiation. At φ = 0%, the microrobot swims at an average speed of 106 ± 17 μm/s. This speed increases to 115 ± 15.2 μm/s at concentration of 0.78 ± 0.1% and 123.3 ± 5.9 μm/s at 1.73 ± 0.11%, as shown in Figure 8A. At actuation frequency of 1 Hz, *Sp* is <2.1 for φ ≥ 0, and increases with the concentration. Our RFT-based model also predicts an increase in the swimming speed with the concentration of the particles. The model predicts speeds of 79.5 and 88.7 μm/s for φ = 0.5% and φ = 2.5%, respectively. Similar behaviour is observed at actuation frequency of 2 Hz (Figure 8B). The microrobot swims at an average speed of 219 ± 20 μm/s at φ = 0%, and the speed increases to 228.9 ± 20.1 μm/s at φ = 0.21 ± 0.2%. At actuation frequency *f* = 3 Hz, the forward speed of the soft microrobotic sperm increases for 0 < φ < 0.53% and decreases with the concentration. This behavior is in a quantitative and qualitative agreement with the theoretical prediction of the RFT-based model, as shown in Figure 8C. At actuation frequencies of *f* = 4 Hz and *f* = 5 Hz, the average swimming speed is measured as 334 ± 104 μm/s and 394 ± 43 μm/s, respectively, for φ = 0% (Figures 7D,E). We attribute the relatively large deviation in swimming speed to the variability in the initial positions of the immersed particles after each trial. A flow-field is created by the flagellum (Figure 3) and the initial positions and concentration of the immersed particles change for each trial. The measured speed of the microrobotic sperm is only compared to the theoretical prediction of our model. It is also essential to compare the deformation of the tail. The time-dependent deformation along the tail depends on the precise initial conditions of the microrobot (position and orientation) and the initial transient of the electronics of the electromagnetic driving system. In addition, the geometric aberrations along the elastic tails and the parameters (magnetization and bending stiffness) entered to the model hinders our effort to obtain quantitative agreement between the predicted time-dependent behavior and measured deformations. Therefore, the time-averaged velocity of the microrobots is only used to compare experimental results to theoretical predictions.

Figure 7F shows the Sperm number of the soft microrobot at various actuation frequencies vs. the concentration of the immersed particles. For *f* = 1 Hz and *f* = 2 Hz, the Sperm number is enhanced by increasing the concentration of the particles. Nevertheless, the optimal Sperm number (associated with maximum propulsive force) is not achieved for concentration range of 0 ≤ φ ≤ 5%. Optimal propulsive force is obtained at actuation frequency of 3 Hz and concentration of 2.4%. As the actuation frequency increases, we observe that the maximum propulsive force is achieved at relatively lower concentrations, and the swimming speed of the microrobot decreases with the concentration of the particles.

In another set of experimental results, the frequency response of a soft microrobotic sperm with relatively longer flexible tail is characterized, as shown in Figure 8. The length of the tail is increased to 346 μm (2*a* = 125 μm and 2*b* = 44 μm). The propulsive force is expected to decrease as the length of the tail increases (as *Sp* becomes >2.1). Figure 8A shows the response of the soft microrobot for 0 < φ < 5% under the influence of oscillating magnetic field at *f* = 1 Hz. The speed of the soft microrobotic sperm is 45 ± 5 μm/s for φ = 0% and increases with the concentration of the particles. The swimming speed is measured as 45.3 ± 5.3 μm/s and 53.6 ± 2.7 μm/s for φ = 0.27 ± 0.23% and φ = 1.73 ± 0.2%, respectively. For 0 < φ < 5% and at *f* = 1 Hz, the propulsion enhancement is achieved owing to the increase in the Sperm number (*Sp* → 2.1) with the concentration. Our RFT-based model also predicts a swimming speed of 31.8 and 47.1 μm/s at φ = 0.27% and φ = 1.73%, respectively. Similarly to actuation frequency of 1 Hz, at *f* = 2 Hz the swimming speed is increased to 60 ± 30 μm/s for φ = 0%, and the propulsion is enhanced for 0 < φ < 10%, as shown in Figure 8B. At *f* = 3 Hz, the speed of the soft microrobot is measured as 121 ± 15 μm/s, 157.5 ± 62.2 μm/s, and 178.5 ± 44 μm/s for φ = 0%, φ = 1.4 ± 0.12%, and φ = 3.6 ± 0.6%, respectively. At these concentrations, the RFT-based model predicts swimming speeds of 182.8 μm/s and 189.1 μm/s at φ = 1.4% and φ = 3.6%, respectively, as shown in Figure 8C. For actuation frequencies *f* > 3 Hz, the increased concentration shifts the Sperm number away from its optimal value (*S*_*p*_ ≈ 2.1). Nevertheless, propulsion is enhanced for 0 < φ < 2.5% and 0 < φ < 4% at *f* = 4 Hz (Figure 8D) and *f* = 5 Hz (Figure 8E), receptively.

Table 1 shows the response of four different soft microrobotic sperm samples to a periodic magnetic field in a fluid with particle concentrations of 0 and 3%. These soft microrobotic sperm samples differ in geometry. The lengths of their flagella are measured as 298, 345, 272, and 286 μm, respectively. Measurements of their swimming speeds indicate that propulsion enhancement is achieved owing to the immersed obstacles in the medium. Gray and Hancock (1955) have shown that positive thrust force can only be achieved if *c*_n_(α) > *c*_t_(α) based on (6). Equations (2) and (4) indicate that the concentration of the immersed particles influences the ratio between the normal and tangential drag coefficients. This dependency provides additional explanation to the behavior of the soft microrobotic sperm samples in a medium with immersed particles. Our experimental results and simulations suggest that planar flagellar propulsion of soft artificial swimmers is enhanced at relatively low actuation frequencies. This behavior implies that the swimming velocity of these artificial swimmers is less likely to be affected in complex and crowded environments at relatively low frequencies of the beating flagellum.

**Table 1:** The forward speed (*v*_*x*_) of a soft microrobotic sperm sample is measured (in μm/s) vs. the concentration (φ) of spherical particles and the actuation frequency (*f*) of the magnetic field.

**φ**	**Microrobot 1**		**Microrobot 2**		**Microrobot 3**		**Microrobot 4**	
	**0%**	**3%**	**0%**	**3%**	**0%**	**3%**	**0%**	**3%**
*f* = 1 Hz	1.2 ≤ *Sp* ≤ 1.7		1.3 ≤ *Sp* ≤ 2		1.1 ≤ *Sp* ≤ 1.6		1.1 ≤ *Sp* ≤ 1.7	
	106 ± 17	105 ± 21	45 ± 5	46 ± 10	56 ± 8	82 ± 8	73 ± 4	85 ± 10
*f* = 2 Hz	1.4 ≤ *Sp* ≤ 2.0		1.6 ≤ *Sp* ≤ 2.4		1.3 ≤ *Sp* ≤ 1.9		1.3 ≤ *Sp* ≤ 2.0	
	228 ± 20	212 ± 44	93 ± 30	124 ± 27	51 ± 10	68 ± 7	157 ± 10	142 ± 28
*f* = 3 Hz	1.6 ≤ *Sp* ≤ 2.3		1.8 ≤ *Sp* ≤ 2.6		1.4 ≤ *Sp* ≤ 2.1		1.5 ≤ *Sp* ≤ 2.2	
	259 ± 33	369 ± 59	151 ± 34	178 ± 44	58 ± 5	81 ± 13	175 ± 55	193 ± 35
*f* = 4 Hz	1.7 ≤ *Sp* ≤ 2.4		2 ≤ *Sp* ≤ 2.8		1.5 ≤ *Sp* ≤ 2.3		1.6 ≤ *Sp* ≤ 2.4	
	334 ± 104	435 ± 30	149 ± 11	203 ± 26	88 ± 7	110 ± 7	160 ± 101	260 ± 17
*f* = 5 Hz	1.8 ≤ *Sp* ≤ 2.6		2.1 ≤ *Sp* ≤ 3.0		1.6 ≤ *Sp* ≤ 2.4		1.7 ≤ *Sp* ≤ 2.5	
	394 ± 43	520 ± 65	198 ± 5	268 ± 48	65 ± 8	82 ± 8	198 ± 61	246 ± 26

*Microrobot 1: 2a = 90 μm, 2b = 48 μm, and L = 298 μm. Microrobot 2: 2a = 125 μm, 2b = 44 μm, and L = 345 μm. Microrobot 3: 2a = 106 μm, 2b = 26 μm, and L = 272 μm. Microrobot 4: 2a = 62 μm, 2b = 59 μm, and L = 286 μm*.

## 4. Conclusions

Like various microorganisms, the propulsion of soft microrobotic sperm samples is enhanced with the concentration of the immersed particles in a viscous heterogenous medium. A hydrodynamic model of the microrobotic sperm is developed based on the RFT to predict the deformation of its tail and the swimming velocity for various concentrations and actuation frequencies. Our simulation results and experiments show that the pressure field created in close proximity to the beating tail is greater than that near to the head at relatively low actuation frequencies (Figure 3). This pressure gradient results in propulsion enhancement at low actuation frequencies and speed reduction at high frequencies as the concentration of the particles increases. The amplitude of the tip of the flagellum is relatively high at low actuation frequencies (Figure 4C) and allows the immersed particles to create a pressure gradient that enhances the propulsion. At relatively high actuation frequency, this amplitude decreases and the pressure field created by the immersed particles is not projected onto the tail and does not improve the propulsion. Despite the relatively large deviations in the measured swimming speeds, our experimental results show mostly quantitative agreement with the theoretical prediction of the RFT-based model. In particular, at relatively low actuation frequencies (*f* = 1 Hz), the measured and calculated swimming speeds increase from 96.4 ± 23.6 μm/s and 79.5 μm/s to 105.3 ± 20.7 μm/s and 88.7 μm/s for φ = 0.5% and φ = 2.5%, respectively. At relatively high actuation frequency (*f* = 5 Hz), the measured and calculated swimming speeds decrease from 394.2 ± 42.8 μm/s and 330.5 μm/s to 352.6 ± 156.2 μm/s and 306.7 μm/s for φ = 0.3% and φ = 2.5%, respectively. A similar phenomenon has been observed in experimental and model observations on motile bacteria in polymer solutions, in which the obstacles consist of semiflexible filaments (Zöttl and Yeomans, 2019). A swimming speed enhancement by a few percent is observed, followed by a reduction in speed, when the volume ratio of polymer filaments is increased. Thus, the experimental observation of enhanced speed of the microrobotic sperm might occur in a small range of particle concentration.

## Author Contributions

IK wrote the paper, conceived the experiments, and analyzed the data. AK designed the simulation results. YH fabricated the robots and conducted the experiments. VM wrote the paper and analyzed the data. MT conducted the experiments. SM participated in drafting the paper and revising it critically.

### Conflict of Interest Statement

The authors declare that the research was conducted in the absence of any commercial or financial relationships that could be construed as a potential conflict of interest.
